# Assessment of Knowledge, Attitude, and Practice about Biomedical Waste Management and Associated Factors among the Healthcare Professionals at Debre Markos Town Healthcare Facilities, Northwest Ethiopia

**DOI:** 10.1155/2018/7672981

**Published:** 2018-10-02

**Authors:** Teshiwal Deress, Fatuma Hassen, Kasaw Adane, Aster Tsegaye

**Affiliations:** ^1^Unit of Quality Assurance and Laboratory Management, School of Biomedical and Laboratory Sciences, College of Medicine and Health Sciences, University of Gondar, P.O. Box 196, Gondar, Ethiopia; ^2^Department of Medical Laboratory Sciences, School of Allied Health Sciences, College of Health Sciences, Addis Ababa University, Addis Ababa, Ethiopia

## Abstract

**Background:**

Healthcare activities restore health and save lives at the same time; however, they can generate hazardous biomedical wastes to a human being or the environment. Generation and disposal of biomedical wastes have become an emerging problem worldwide.

**Objective:**

To assess knowledge, attitude, and practice about biomedical waste management and associated factors among healthcare professionals in Debre Markos town healthcare facilities, northwest Ethiopia.

**Methods:**

A cross-sectional study was employed, and data were collected through structured self-administered questionnaire and observational checklist. Data were entered into the Epi-data 3.1 software and exported into SPSS version 20 for analysis. Bivariate and multivariate logistic regression analyses were computed. Variables with a *P* value of <0.05 in the multivariate logistic regression analysis were considered to explain the presence of statistically significant associations.

**Result:**

Among 296 healthcare professionals studied, 168 (56.8%), 196 (66.2%), and 229 (77.4%) had adequate knowledge, favorable attitude, and adequate practice score, respectively. Regarding associated factors, MSc and MD^+^ (AOR: 4, 95% CI: (1.37, 149.52)), BSc holders (AOR: 2.53, 95% CI: (1.47, 4.38)), and availability of color-coded bins (AOR: 7.68, 95% CI: (3.30, 17.89)) were identified more likely to contribute for adequate knowledge, favorable attitude, and adequate practice scores, respectively.

**Conclusion:**

The level of knowledge, attitude, and practice scores were not satisfactory. Majority of the healthcare professionals did not access biomedical waste management training. Educational level, use of visual aid, and availability of color-coded bins in the department were identified as a factor for biomedical waste management. Regular training should be given to healthcare professionals.

## 1. Background

During the healthcare delivery process, healthcare establishments can inevitably generate hazardous biomedical wastes (BMWs) to a human being or the environment [1]. These wastes are broadly categorized as general (nonhazardous) and hazardous waste. General waste is constituted 85% of the total waste while the remaining 15% is a hazardous waste [2]. There are currently several terms used to describe wastes generated from the healthcare establishments such as clinical waste, healthcare waste, infectious waste, medical waste and biomedical waste are typically encountered [3]. However, BMW is the most frequently used term in most articles. So, in this study, we used this term to represent wastes originated from the healthcare facilities (HCFs). Biomedical waste is generated during diagnosis, treatment or immunization of human beings which mainly includes syringes, needles, ampoules, dressing materials, disposable plastics, and microbiological wastes [4]. The main sources of BMWs are hospitals and primary healthcare facilities [5].

The appropriate biomedical waste management (BMWM) process includes vital steps (segregation, storage, transportation, treatment, and disposal) [6, 7] which requires special attention [2, 8–10]. The World Health Organization has prepared a BMWM guideline to ensure safe management of the wastes from the HCFs [2]. In addition, there are three main BMWM guidelines in Ethiopia [9–11]. According to these guidelines, as a minimum option, HCFs are required to segregate (separate) BMWs using three types of color-coded bins (black, yellow, and safety box) [9–11].

Generation and disposal of BMWs have become an emerging problem worldwide [8]. It has become a major cause of concern for HCFs and the environment [12, 13]. According to the World Health Organization report, 10–25% of the BMW was estimated to be hazardous [2]. However, evidence from different kinds of literatures indicated that the proportion of hazardous waste is varied from country to country ranging from 20% to 75% including Ethiopia [14–21]. Biomedical waste management is still at the infancy stage and recently got attention [22] due to increased awareness about human immunodeficiency virus, hepatitis B virus, hepatitis C virus, and other potentially infectious diseases [23]. Biomedical wastes can transmit more than 30 dangerous bloodborne pathogens [24].

A systematic review of 150 articles published since 2000 revealed that at least 50% of the world population is threatened by public health risks due to mismanagement of BMWs [25]. This is because any carelessness of BMWM can spread infections and contaminate the entire environment [13]. All individuals, particularly healthcare professionals (HCPs), who are on the first line of contact are potentially at risk [26]. Especially, poor BMWM is a problem in most developing countries due to lack of awareness and trained clinical staff in the waste management framework. In addition, the absence of BMWM guideline and suitable treatment and disposal methods could further obstruct the waste management efforts [3]. Currently, BMW disposal in HCFs has become an increasing issue [27] and credible evidence showed that BMWM across Ethiopian health institutions is still inadequate [20]. It was a neglected activity by the healthcare providers and lacked attention it deserves [10].

Biomedical waste management and evaluation studies conducted so far in different parts of Ethiopia reported a high proportion of hazardous BMW generation rates. However, little was reported about the level of knowledge, attitude, practice scores, and associated factors among HCPs who have key roles to ensure effective BMWM. Measuring the level of knowledge, evaluating the attitude, and assessing the practice of healthcare providers and associated factors on BMWM are the key objectives to consider for safe healthcare practice. Therefore, this study is aimed at filling this gap and recommending policymakers to design and implement appropriate intervention to improve safe BMWM in HCFs.

## 2. Materials and Methods

### 2.1. Study Area and Setting

The study was conducted in Debre Markos town which is located in Amhara regional state at a distance of 300 kilometers from Addis Ababa to the northwest and 265 kilometers from Bahir Dar to the southeast. According to the Ethiopian Central Statistical Agency (CSA) report [28], the town has a population of 119,000. Among these, 97.1%, 1.7%, and 1.1% were Orthodox Christian, Muslim, and Protestant Christian religion followers, respectively. The three largest ethnic groups in the town were Amhara (97.12%), Tigrinya (1.29%), and Oromo (0.67%) [29]. One referral hospital, 4 health centers, and 12 clinics are found within the town. The hospital has 5 inpatient wards (gynecological, surgical, medical, pediatric, and eye unit) with a total of 400 beds. During 2016/17, the hospital has 35 doctors, 144 nurses, 25 midwives, and 33 laboratory professionals and currently serves 5 million inhabitants in its catchment area [30]. Health centers and clinics have 57 nurses, 24 health officers, 23 laboratory professionals, and 11 midwives, and they currently provide basic health services for the town and nearby areas.

### 2.2. Study Design and Period

An institution-based cross-sectional study was employed from November 2016 to June 2017.

### 2.3. Sample Size and Sampling Technique

A total of 296 HCPs were studied from 12 HCFs (1 hospital, 4 health centers, and 7 clinics) after excluding those study participants who did not fulfil the eligibility criteria. For this study, an entire population sampling technique (survey) was employed. This sampling technique was used because the number of the study population in the HCFs was small which is manageable for data collection and analysis.

### 2.4. Eligibility Criteria

All HCPs of the five health professions (medical, laboratory, nurse, health officer, and midwifery) who have at least one year of work experience and permanently employed among Debre Markos town HCFs were studied. Study participants who were available during the data collection period and willing to take part in the study were included. These HCPs were selected because they are mainly involved in the generation, segregation, and management of BMWs than other HCPs. They frequently handle/work with high-risk BMWs who become at risk of occupational health hazards and play a key role to protect the community.

### 2.5. Data Collection Tools and Procedure

#### 2.5.1. Data Collection Tools

Structured self-administered questionnaire and observational checklist were used to collect the data. The questionnaire was developed through a review of the available scientific literatures and national [9–11] and international [2] BMWM guidelines. The questionnaire consisted of four sections (sociodemographic and HCF related factors, knowledge, attitude, and practice).

#### 2.5.2. Data Collection Procedure

Two trained data collectors (medical laboratory technologist and clinical nurse) were assigned for the data collection process, and the medium of language for data collection was English. Written informed consent containing questionnaires was distributed as hard copy for the study participants. Then data collectors collected completed questionnaires from the study participants, and questionnaires were checked for completeness. Incomplete questionnaires were taken back to the study participants for completion. After completion of the questionnaires, data collectors filled individual observational checklists while the study participants were providing healthcare services. Finally, observational checklists and questionnaires were labeled with HCF and individual identification code numbers and attached together accordingly. After completion of all questionnaires and individual observational checklists, facility observation was conducted using the predetermined structured observational checklist.

#### 2.6. Methods of Measurement (Scoring)

All questions of the questionnaire and observational checklist were close-ended. The questionnaire consisted of knowledge, attitude, and practice domains.

*(1) Knowledge Domain*. This domain consisted of 21 multiple-choice questions and each question had either three or four possible options. Knowledge questions were scored either “1” or “0 for the correct and incorrect response, respectively.” The total knowledge score for each study participant was computed, and the possible score could range from 0 to 21. Then, the overall knowledge score was computed by summation of all the individual study participants' total knowledge scores. Finally, the mean score was calculated by dividing the overall knowledge score by the number of study participants (296). Knowledge scores below and above or equal to the mean score were assigned for inadequate and adequate knowledge, respectively [23, 31].

*(2) Attitude Domain*. It comprised of 16 Likert items. A five-point Likert scale of measurement was used to represent scores, as such “Strongly Disagree,” “Disagree,” “Neutral,” “Agree,” and “Strongly Agree” and were given numerical scores 1, 2, 3, 4, and 5, respectively. For negatively phrased statements, scores were reversely coded during the data entry period as 5, 4, 3, 2, and 1. Then, the composite score for each study participant was computed which could range from 16 to 80. The overall attitude score was calculated by adding all the study participants' attitude scores, and the mean score was computed by dividing the overall attitude score by the number of study participants (296). Finally, attitude scores below the mean and above or equal to mean score were assigned for unfavorable and favorable attitude, respectively [31].

*(3) Practice Domain*. Nine multiple-choice practice questions were used, and they were dichotomized by giving “1” or “0” point for correct and incorrect responses, respectively. Likewise, knowledge and attitude questions, for practice questions total individual and overall practice scores were calculated. Then, the mean score was calculated by dividing the overall practice score by the number of study participants (296). Practice scores below the mean and above or equal to the mean score were assigned for inadequate and adequate practice, respectively [31].

#### 2.7. Data Quality Control

Data collection tools were validated with 10% of the study population who were not included in the final study. According to the pilot survey, the contents of the data collection tools were slightly modified and suggestions from different persons were included. Training was given for data collectors. Timely supervision of data collectors was done by the investigators.

#### 2.8. Data Management and Analysis

Data were entered into Epi-data 3.1 software and then exported to SPSS (Statistical Package for Social Sciences) version 20 for analysis. Descriptive statistics were calculated through cross-tabulation. Bivariate and multivariate logistic regression analyses were computed to identify predictor variables with the statistically significant association. A standard (Enter) method was used for variable selection for the multivariate logistic regression analysis, which means all variables with a *P* value of ≤0.2 in the bivariate analysis were pooled into the multivariate logistic regression analysis. Variables with a *P* value of <0.05 in the multivariate logistic regression analysis were used to explain the presence of statistically significant associations between the predictor and outcome variables. Finally, Odds Ratio (OR) with 95% confidence interval (CI) was used to determine the strength of association.

#### 2.9. Ethical Considerations

Ethical approval was obtained from the Departmental Research and Ethics Review Committee (DRERC) of the Department of Medical Laboratory Sciences, Addis Ababa University. Official letters were written from east Gojjam Zonal health department to HCFs, and permission was obtained from the HCFs.

## 3. Results

### 3.1. Sociodemographic and HCF-Related Characteristics

Two hundred ninety-six study participants were included from 12 HCFs. Among these, 197 (66.6%), 69 (23.3%), and 30 (10.1%) were from the hospital, health centers, and clinics, respectively. The mean age of the study participants was 30.46 ± 6.64 years. Less than one-third (30.7%) of the study participants were vaccinated for hepatitis B virus. Regarding previous training, only 109 (36.8%) had taken BMWM training. One hundred seventy-seven (59.8%) of the study participants got information from the guideline. Sixty-nine (23.3%) of the study participants had encountered needlestick/sharps injuries preceding 12 months of the data collection period. Most (97%) of the HCPs respond the availability of sufficient quantity gloves and 81.4% of the respondents also disclosed the availability all types (black, yellow, and safety box) of color-coded bins in their department (Table 1).

### 3.2. Knowledge, Attitude, and Practice of Study Participants

#### 3.2.1. Knowledge of Study Participants

In this study, HCPs with adequate knowledge score were 168 (56.8%). One hundred sixty-nine (57.1%) of the study participants identified the biohazard symbol. Regarding knowledge on segregation of BMWs, 235 (79.4%), 217 (73.3%), and 253 (85.5%) of the study participants were aware that general, infectious, and sharp wastes should be placed in a black, yellow, and a safety box, respectively. In addition, 254 (85.8%) of them were aware that a safety box should be filled only a maximum of 3/4th. Only twenty-nine (9.8%) of the study participants knew the maximum storage time of infectious wastes before treatment or disposal. Two hundred eighteen (73.6%) of them knew 72 hours as a maximum time delay to start HIV postexposure prophylaxis. All doctors were concerned about needlestick injury than other healthcare professionals. About 46% of health officers did not consider all BMW as hazardous (Table 2).

#### 3.2.2. Attitude of Study Participants

One hundred ninety-six (66.2%) of the study participants had favorable attitude score on BMWM. The mean attitude score of Likert items ranged from 3.80 to 4.45. In addition, 161 (45.9%) of the study participants strongly agreed to the statement “BMWs should be segregated into different categories at the source” and 191 (58.1%) study participants agreed to the statement “safe BMWM is an issue involving a teamwork.” However, to make similar attitude score category with other studies, the five-point Likert scale of measurement was categorized into a three-level Likert scale. Which means strongly disagree and disagree were merged and labeled as disagree; similarly, strongly agree and agree were merged and labeled as agree, whereas neutral remained as it was (Table 3).

#### 3.2.3. Practice of Study Participants

In this study, 229 (77.4%) of the study participants had adequate practice score and 174 (58.8%) used a visual aid in their department/section. Regarding the use of personal protective equipment, 277 (94%) and 288 (97%) of the study participants have always used gloves and gown, respectively, while they were handling BMWs. Two hundred eighty-eight (79.1%) of the study participants practiced labeling BMW containers. With respect to segregation of BMWs, 275 (92.9%) of the study participants segregated BMWs at the source of generation. However, only 261 (88.2%) of them followed color coding segregation. Among these, 228 (77%), 198 (66.9%), and 247 (83.4%) of them put general, infectious, and sharp wastes into the black bin, yellow bin, and safety box, respectively. More specifically, 26 (83.9%), 140 (85.9%), 27 (100%), 45 (91.8%), and 23 (88.5%) doctors, nurses, midwives, laboratory professionals, and health officers, respectively, followed color coding segregation.

#### 3.2.4. Observational Result

Slightly above three-fourths (76%) of the study participants practiced BMW segregation at the source, and 225 (75%) of them used biohazard symbol-labeled safety boxes for sharp waste segregation. In this study, 70 (23.6%) and 69 (23.3%) of the study participants were working with yellow and black bins containing mixed wastes, respectively. Similarly, one-fourth (25%) of the study participants were working with at least one unlabelled BMW containers, and 65 (22%) HCPs were observed using more than 3/4th filled infectious waste containers. Regarding HCF observation, most (91.7%) of them used puncture-resistant bins to store BMWs temporarily, whereas the other used the incinerator chamber. All HCFs treated BMWs on-site. Among these, 11 (91.7%) used incineration and the remaining used open burning. From the empirical observation, most nongovernmental healthcare facilities' incinerators had remnants of incompletely burned BMWs. Most (91.7%) of the HCFs disposed of the ash in the placenta pit, latrine opening, or open ground.

### 3.3. Associated Factors

#### 3.3.1. Factors Associated with Knowledge

In the bivariate analysis, age group, job category, and information sourced from the guideline were marginal, whereas educational level, presence of BMWM committee, working experience, previous training, presence of BMWM guideline, attitude, and practice scores of study participants showed a statistically significant association with the knowledge score. After adjustment of possible confounds, however, MSc and MD^+^ (AOR: 14, 95% CI: (1.37, 149.52)), working in another department (AOR: 2.22, 95% CI: (1.03, 4.77)), attitude score (AOR: 2.09, 95% CI: (1.09, 4.00)), and practice score (AOR: 2.28, 95% CI: (1.18, 4.42)) of the study participants were more likely to contribute for adequate knowledge score compared with the respective reference groups given that other predictor variables were held constant (Table 4).

#### 3.3.2. Factors Associated with Attitude

In the bivariate analysis, sex, and type of HCF were marginal, whereas information source from the guideline, educational level, previous training, and knowledge scores of the study participants showed a statistically significant association with the attitude score. After adjustment of possible confounds, however, information source from guideline (AOR: 1.82, 95% CI: (1.07, 3.10)) and BSc holders (AOR: 2.53, 95% CI: (1.47, 4.38)) was more likely to be contributed for favorable attitude score compared with the reference groups given that other predictor variables were held constant (Table 5).

#### 3.3.3. Factors Associated with Practice

In the bivariate analysis, working department and job category of study participants were marginal, whereas the presence of guideline, educational level, previous training, use of visual aid, and presence of color-coded bins in the department showed a statistically significant association. After adjustment of possible confounds, use of visual aid and availability of all the three types of color-coded bins in the department (AOR: 5.34, 95% CI: (2.87, 9.95), and AOR: 7.68, 95% CI: (3.30, 17.89), respectively) were more likely to contribute to adequate BMWM practice given that other predictor variables were constant (Table 6).

## 4. Discussion

Healthcare facilities have a responsibility to protect the environment and public health. Thus, providing training for HCPs for effective BMWM is a very critical step. However, in this study, only 36.8% of the study participants were trained on BMWM which is lower than 61.6% and 46.9% studies conducted in Bangladesh and Gondar town, respectively [32, 33]. This result was, however, more or less similar to a study conducted in Adama, Ethiopia 31% [16].

Regarding incidence of needlestick/sharp injuries, about 23.3% cases occurred during the previous 12 months preceding the data collection time, which is better than 51% and 30.8% studies conducted in Nigeria and Gondar town, respectively [34, 35]. However, a similar study (25%) was found in Gondar town with a different time period [35].

According to the World Health Organization and Ethiopian Food, Medicine and Healthcare Administration and Control Authority BMWM guidelines, to prevent occupational health risks, healthcare workers should be protected by hepatitis B virus vaccination [2, 9]. However, in this study, hepatitis B virus vaccination was low (30.7%) as the level of occupational exposure among HCPs is high. It is extremely low compared to 85.8% and 95% studies conducted in India and Iran, respectively [13, 36]. Unavailability and cost of the vaccine could be the possible causes for low vaccination status of HCPs in the current study.

### 4.1. Knowledge of Study Participants

Adequate knowledge is vital for appropriate BMWM practice. However, in this study, only 56.8% of the study participants had adequate knowledge score, which is better than 45% and 40.5% studies conducted in Nigeria and Sri Lanka, respectively [37, 38]. A better result was found in Pakistan where 96% of the study participants had good knowledge score [39].

This could be due to the difference in availability and utilization of waste management guidelines among the facilities, providing training opportunity for HCPs, national health sector strategy difference, or it might be due to academic performance difference of study participants. As a minimum standard, a three-bin system of BMW segregation has been established in Ethiopia [9, 10]. However; only 77.2% of the study participants had knowledge of color coding segregation which is lower than 92.3% of a report from India [31]. About 72.6%, 78.3%, and 86.3% study participants were able to identify that general, infectious, and sharp wastes should be placed in black, yellow, and a safety box, respectively. According to guidelines, infectious waste containers should be labeled with a biohazard symbol [2, 9–11]. However, only half (53.6%) of study participants were able to identify the biohazard symbol which is similar to a study in India (54.4%) [40]. However, a better result was found in Nainital city in India where the majority of HCWs (85.5%) were able to identify a symbol of biohazards [23].

### 4.2. Attitude of Study Participants

The overall favorable attitude score of HCPs was 62.1% which is more or less comparable with 59.9% in a study conducted at Gondar town [41]. Similarly, the majority of study participants in Sri Lanka and almost all studied participants in the Tripura state of India had favorable attitude [31, 38]. However, this study was better than a study from Nigeria [37]. This could be due to methodological difference or commitment of healthcare staff for waste management. With regard to waste segregation and treatment, about 86.3% and 74.6% study participants agreed that BMWs should be segregated at the source and disinfected before disposal, respectively. A similar study was found in India in which about 88.1% study participants agreed on segregation of BMWs at the source [31].

### 4.3. Practice of Study Participants

Adequate practice score of the study participants was 78.9%, which is better than 31.5% and 74.8% in studies conducted in Ethiopia and Sri Lanka [33, 38], respectively. However, a better result was found from Pakistan where 94.3% of the study participants had adequate practice [39]. This could be due to lack of training, HCPs commitment, motivation, and enforcement from concerned bodies or ignorance of HCPs for BMWM. The highest practice score was noted among midwives (92.6%); however, the list was disappointingly among medical doctors (58.1%). One could ask if over qualification leads to ignorance. However, it is more or less comparable to a study from Bangladesh, where 44% of medical doctors studied had adequate practice [42]. Similarly, a study conducted in India indicated that the highest adequate practice score was among Nurses (97.3%) followed by doctors (77.8) [43]. Probably this difference could be due to the accessibility of BMWM equipment, training opportunity, and guidelines. Biomedical waste segregation is the most critical step for proper waste management, and it should be done at the point of generation using color-coded bins [2]. All hazardous wastes should be segregated at the point of generation [2,9–11]; however, in this study, only 88.2% of HCPs were segregated at the source at the source of generation.

### 4.4. Treatment and Disposal

Most studied HCFs (91.7%) used puncture-resistant containers for BMW storage until treatment or disposal, and the remaining used the incinerator chamber as a temporary waste storage means. These practices are not in line with the national guideline requirement where all HCFs should have separate waste storage facilities for hazardous BMWs [10]. In most HCFs, waste treatment was done according to the volume of waste collected rather than the time of storage [10]. Two HCFs (16.7%) burn all types of BMWs in an unprotected environment. Most HCFs (91.7%) did not have specifically designed ash pit, and they dispose of either in placenta pit, latrine opening, or open dumping. These are bad activities which are strongly prohibited and are out of the guidelines recommendation [2, 9, 10].

### 4.5. Limitations of the Study

In this study, liquid BMWs were not assessed due to financial constraint. Since the study was conducted in a limited geographical area, it could not be generalized at a national level. Similarly, BMW generation rates among studied facilities were not measured due to financial constraint. Healthcare facility observation was conducted at one point in time, which may have an implication of the study.

## 5. Conclusion and Recommendation

In this study, HCPs level of knowledge, attitude, and practice scores is low. The majority of the studied HCPs did not access BMWM training. Similarly, about half of them did not access BMWM guidelines in their department. Educational level, use of visual aid, and availability of all the three types of color-coded bins in the department/working section were identified as a key factor for effective BMWM. Regular waste management training should be given for HCPs, and they should have access to BMWM guidelines in their department/healthcare delivery section. In addition, periodic and comprehensive studies should be conducted.

## Figures and Tables

**Table 1:** Sociodemographic and HCF-related factors for BMWM at Debre Markos town HCFs, 2017 (*n*=296).

Sociodemographic and HCF-related variables	Variable category	Study participant, *n* (%)
Gender	Male	177 (59.8)
	Female	119 (40.2)

Age of respondents	≤25 years	44 (14.9)
	26–30 years	159 (53.7)
	31–35 years	42 (14.2)
	>35 years	42 (14.2)
	Missing	9 (3)

Educational level	MSc and MD^+^	20 (6.8)
	BSc	170 (57.4)
	Diploma	106 (35.8)

Job category	Medical doctor	31 (10.5)
	Nurse	163 (55.1)
	Midwife	27 (9.1)
	Laboratory professional	49 (16.6)
	Health officer	26 (8.8)

Working department/section #	OPD	102 (34.5)
	Ward	93 (31.4)
	Laboratory room	48 (16.2)
	Emergency	64 (21.6)
	Others	72 (24.3)

Work experience	1–5 years	143 (48.3)
	6–10 years	98 (33.1)
	>10 years	49 (16.6)
	Missing	6 (2)

Working hours per day	<8 hours	6 (2)
	8 hours	249 (84.1)
	>8 hours	35 (11.8)
	Missing	6 (2)

Availability of waste management guideline	Yes	159 (51.6)
	No	101 (34.1)
	Not sure	36 (12.2)

Availability of BMWM committee in the facility	Yes	188 (63.5)
	No	57 (19.3)
	Not sure	51 (17.2)

# denotes multiple response question; MD^+^: medical specialists.

**Table 2:** Frequency of study participants among each knowledge item question at Debre Markos town HCFs, 2017 (*n*=296).

Variables	Job category, *n* (%)				
	Doctor (*n*=31)	Nurse (*n*=163)	Midwife (*n*=27)	Laboratory (*n*=49)	Health officer (*n*=26)
Does your facility generate BMWs?	24 (77.4)	129 (79.1)	24 (88.9)	43 (87.8)	24 (92.3)
Do you know about BMWM?	24 (77.4)	113 (69.3)	20 (74.1)	38 (77.6)	24 (92.3)
Is there any health hazard associated with BMWs?	30 (96.8)	148 (90.8)	24 (88.9)	43 (87.8)	24 (92.3)
Is needlestick/sharp injury a concern?	31 (100)	147 (90.2)	25 (92.6)	43 (87.8)	25 (96.2)
Does wearing personal protective equipment reduce the risk of infection?	29 (93.5)	149 (91.4)	26 (96.3)	47 (95.9)	25 (96.2)
Are all BMWs hazardous?	23 (74.2)	99 (60.7)	21 (77.8)	40 (81.6)	14 (53.8)
Are body fluid contaminated items considered as BMWs?	31 (100)	134 (82.2)	26 (96.3)	45 (91.8)	23 (88.5)
Do you know about color coding segregation of BMWs?	26 (83.9)	120 (73.6)	21 (77.8)	40 (81.6)	20 (76.9)
Should infectious waste containers be labeled with a biohazard symbol?	24 (77.4)	126 (77.3)	23 (85.2)	35 (71.4)	25 (96.2)
Should BMWs be segregated at the point of generation?	25 (80.6)	137 (84.0)	22 (81.5)	40 (81.6)	24 (92.3)
Does disinfection of BMWs decrease infection transmission?	27(87.1)	159 (97.5)	27 (100)	47 (95.9)	26 (100)
Do we need to close BMW containers while transport?	27 (87.1)	127 (77.9)	21 (77.8)	38 (77.6)	24 (92.3)
Do we need to secure BMWs awaiting treatment/disposal?	26 (83.9)	132(81)	24 (88.9)	39 (79.6)	19 (73.1)
Do you know about BMW disposal methods?	23 (74.2)	98 (60.1)	21 (77.8)	33 (67.3)	22 (84.6)

*n* (%) is the proportion of study participants who correctly answered each knowledge question; BMW: biomedical waste; BMWM: biomedical waste management.

**Table 3:** Frequency distribution of study participants among each Likert item of BWM at Debre Markos town HCFs, 2017 (*n*=296).

Predictor variables	Response options		
	Disagree, *n* (%)	Neutral, *n* (%)	Agree, *n* (%)
Improperly managed BMWs may cause infection	37 (12.5)	7 (2.4)	252 (85.1)
Proper BMW handling is an issue	34 (11.5)	3 (1.0)	259 (87.5)
Safe BMWM need a teamwork	25 (8.4)	12 (4.1)	259 (87.5)
HIV may be transmitted through BMWs	27 (9.1)	1 (.3)	268 (90.5)
HIV postexposure prophylaxis help to prevent the development of HIV infection	32 (10.8)	8 (2.7)	256 (86.5)
HBV may be transmitted through BMWs	14 (4.7)	8 (2.7)	274 (92.6)
HCV may be transmitted through BMWs	41 (13.9)	34 (11.5)	221 (74.7)
BMWs do not transmit any infectious diseases	24 (8.1)	12 (4.1)	260 (87.8)
BMWs should be segregated into different categories at the point of generation	41 (13.9)	14 (4.7)	241 (81.4)
BMW segregation facilitates safe handling	40 (13.5)	8 (2.7)	248 (83.8)
Labelling BMW containers have no significance	52 (17.6)	12 (4.1)	232 (78.4)
Proper BMW disposal is important to prevent infection transmission	23 (7.8)	2 (.7)	271 (91.6)
BMW disinfection can reduce the chance of contracting the infection	32 (10.8)	10 (3.4)	254 (85.8)
Wearing personal protective equipment helps to reduce the risk of infection	25 (8.4)	5 (1.7)	266 (89.9)
BMWM add extra burden of work	83 (28.0)	21 (7.1)	192 (64.9)
Biohazardous wastes should be disinfected before disposal	59 (19.9)	21 (7.1)	216 (73.0)

**Table 4:** Bivariate and multivariate logistic regression analysis of factors against knowledge scores of study participants at Debre Markos town HCFs, 2017 (*n*=296).

Variables			Knowledge		COR (95% CI)	*P* value	AOR (95% CI)	*P* value
			IK (*n*)	AK (*n*)				
Age group		≤25 years	17	27	0.433 (0.17, 1.13)	0.086		
		26–30	48	111	0.63 (0.28, 1.42)	0.265		
		31–35	9	33	1.0 (0.35, 2.84)	1.00		
		≥36 years	9	33	1			

Job category		Doctor	8	23	0.69 (0.19, 2.42)	0.557		
		Nurse	57	106	0.44 (0.16, 1.24)	0.120		
		Midwife	7	20	0.68 (0.19, 2.50)	0.562		
		Laboratory	10	39	0.93 (0.28, 3.08)	0.903		
		Health officer	5	21	1			

Educational level		MSc and MD^+^	1	19	7.85 (1.01, 61.25)	0.049^*∗*^	4 (1.37, 149.52)	0.023^*∗*^
		BSc	55	115	0.86 (0.51, 1.47)	0.588		
		Diploma	31	75	1		1	

Department	Ward	Yes	32	61	0.71 (0.42, 1.20)	0.201		
		No	55	148	1			
	Laboratory	Yes	9	39	1.99 (0.92, 4.31)	0.081		
		No	78	170	1			
	Others	Yes	26	46	0.66 (0.38, 1.16)	0.152	2.22 (1.03, 4.77)	0.042^*∗*^
		No	61	163	1		1	

Work experience		1–5 years	52	91	1			
		6–10 years	23	75	1.86 (1.05, 3.32)	0.035^*∗*^		
		>10 years	10	39	2.23 (1.03, 4.83)	0.042^*∗*^		

Information source	Guideline	Yes	45	132	1.60 (0.97, 2.65)	0.069		
		No	42	77	1			
	Training	Yes	28	114	2.53 (1.50, 4.28)	0.001^*∗*^		
		No	59	95	1			
	Others	Yes	29	36	0.42 (0.24, 0.74)	0.003^*∗*^		
		No	58	173	1			

Previous training		Yes	20	89	2.49 (1.41, 4.39)	0.002^*∗*^		
		No	67	120	1			

Presence of BMWM committee in the facility		Yes	45	143	2.02 (1.21, 3.37)	0.007^*∗*^		
		No and not sure	42	66	1			

Presence of guideline		Yes	32	127	2.66 (1.59, 4.46)	0.001^*∗*^		
		No and not sure	55	82	1			

Attitude score		Unfavorable	38	62	1		1	
		Favorable	49	147	1.84 (1.10, 3.08)	0.021^*∗*^	2.09 (1.09, 4.00)	0.026^*∗*^

Practice score		Inadequate	41	66	1		1	
		Adequate	46	143	1.93 (1.16, 3.22)	0.012^*∗*^	2.28 (1.18, 4.42)	0.014^*∗*^

AK: adequate knowledge; IK: inadequate knowledge; MD^+^: medical specialists; MSc: master of science; BSc: bachelor of science; COR: crude odds ratio; AOR: adjusted odds ratio; CI: confidence interval; ^*∗*^statistically significant at *P* value <0.05.

**Table 5:** Bivariate and multivariate logistic regression analysis of factors against attitude scores of study participants at Debre Markos town HCFs, 2017 (*n*=296).

Variables		Attitude		COR (95% CI)	*P* value	AOR (95% CI)	*P* value
		UA (*n*)	FA (*n*)				
Sex	Male	52	125	1.63 (.10, 2.65)	0.051		
	Female	48	71	1			

Information source from guideline	Yes	48	129	2.09 (1.28, 3.41)	0.003^*∗*^	1.82 (1.07, 3.10)	0.028^*∗*^
	No	52	67	1		1	

Educational level	MSc and MD^+^	4	16	3.85 (1.21, 12.29)	0.023^*∗*^		
	BSc	44	126	2.76 (1.65, 4.60)	0.001^*∗*^	2.53 (1.47, 4.38)	0.001^*∗*^
	Diploma	52	54	1		1	

Type of facility	Hospital	57	140	1.64 (0.74, 3.62)	0.200		
	Health center	31	38	0.82 (0.34, 1.95)	0.650		
	Clinic	12	18	1			

Training	Yes	27	82	1.2 (0.32, .86)	0.010^*∗*^	1.4 (0.26, 0.80)	0.006^*∗*^
	No	53	134	1		1	

Knowledge score	Inadequate	38	49	0.54 (0.32, 0.91)	0.021^*∗*^	0.44 (0.25, 0.78)	0.005^*∗*^
	Adequate	62	147	1		1	

FA: favorable attitude; UA: unfavorable attitude; MD^+^: medical specialists: MSc: master of science; BSc; bachelor of science; COR: crude odds ratio; AOR: adjusted odds ratio; CI: confidence interval; ^*∗*^statistically significant at a *P* value of <0.05.

**Table 6:** Bivariate and multivariate logistic regression analysis of factors against practice scores of study participants at Debre Markos town HCFs, 2017.

Variables			Practice		COR (95% CI)	*P* value	AOR (95% CI)	*P* value
			IP (*n*)	AP (*n*)				
Working department	OPD	Yes	44	58	0.63 (0.39, 1.04)	0.070		
		No	63	131	1			
	Ward	Yes	28	65	1.48 (0.88, 2.50)	0.144		
		No	79	124	1			
	Laboratory	Yes	22	26	1.48 (0.88, 2.50)	0.144		
		No	85	163	1			

Information source from	Guideline	Yes	58	119	1.44 (0.89, 2.32)	0.141		
		No	49	70	1			
	Training	Yes	41	101	1.85 (0.14, 3.0)	0.013^*∗*^		
		No	66	88	1			
	Others	Yes	31	34	0.54 (0.31 0.94)	0.030^*∗*^		
		No	76	155	1			

Educational level		MSc and MD^+^	6	14	0.92 (0.32, 2.62)	0.877		
		BSc	71	99	0.55 (0.33, 0.93)	0.025^*∗*^		
		Diploma	30	76	1			

Job category		Doctor	16	15	0.50 (0.17, 1.45)	0.20		
		Nurse	50	113	1.2 (0.50, 2.87)	0.687		
		Midwife	9	18	1.10 (0.34, 3.30)	0.922		
		Laboratory	23	26	0.60 (0.22, 1.60)	0.306		
		Health officer	9	17	1			

Previous training		Yes	28	81	2.12 (1.26, 3.55)	0.005^*∗*^		
		No	79	108	1			

Use of visual aid		Yes	35	139	5.72 (3.41 9.59)	0.001^*∗*^	5.34 (2.87, 9.95)	0.001^*∗*^
		No	72	50	1		1	

Presence of BMWM committee		Yes	55	133	2.25 (1.37, 3.67)	0.001^*∗*^		
		No	52	56	1			

Availability of guideline		Yes	44	115	2.23 (1.37, 3.61)	0.001^*∗*^		
		No	63	74	1			

Availability of color-coded bins		Yes	64	177	9.91 (4.92, 20.0)	0.001^*∗*^	7.68 (3.30, 17.89)	0.001^*∗*^
		No	43	12	1		1	

Knowledge group		Inadequate	41	46	0.52 (0.31, 0.86)	0.012^*∗*^		
		Adequate	66	143	1			

AP: adequate practice; IP: inadequate practice; OPD: outpatient department; MD^+^: medical specialists; MSc: master of science; BSc: bachelor of science; COR: crude odds ratio; AOR: adjusted odds ratio; CI: confidence interval; ^*∗*^statistically significant at *P* value <0.05.

## Data Availability

The data used to support the findings of this study are available from the corresponding author upon request.

## Abbreviations

BSc: Bachelor of sciences
HCFs: Healthcare facilities
HCPs: Healthcare professionals
BMW: Biomedical waste
BMWM: Biomedical waste management
OPD: Outpatient department
MSc: Masters of sciences
COR: Crude odds ratio
AOR: Adjusted odds ratio
CI: Confidence interval
AK: Adequate knowledge
IK: Inadequate knowledge
FA: Favorable attitude
UA: Unfavorable attitude
AP: Adequate practice
IP: Inadequate practice.

## Supplementary Materials

Supplementary Materials Questionnaire and observational checklist.
