# Supplementary material for: Assessment of Knowledge, Attitude, and Practice about Biomedical Waste Management and Associated Factors among the Healthcare Professionals at Debre Markos Town Healthcare Facilities, Northwest Ethiopia
Source: J Environ Public Health. 2018 Oct 2;2018:7672981. doi: 10.1155/2018/7672981 (PMC6189693; doi:10.1155/2018/7672981)
Supplement: Supplementary Materials — Questionnaire and observational checklist. [file 7672981.f1.docx]

Appendix

Questionnaire and observational checklist

**Healthcare Facility Identification Code No. ______**

**Study Participant Identification Code No. ______**

**Information sheet**

**Title of the study:** Assessment of Knowledge, Attitude, and Practice about Biomedical Waste Management and Associated Factors among Healthcare Professional at Debre Markos Town Healthcare Facilities, Northwest Ethiopia

**Objective:** The objective of this study is to assess the knowledge, attitude and practice about biomedical waste management and associated factors among healthcare professionals at Debre Markos town healthcare facilities.

**Ethical approval:** The title of the study was approved by the Departmental Research and Ethics Review Committee (DRERC) of the Addis Ababa University.

**Perceived benefits and risk:** Being participated in this study, you may not be benefited personally; however, the result of the study may benefit patients, healthcare providers, healthcare facility managers, researchers, policymakers and other stakeholders as appropriate. Being a participated in this study does not induce any risk.

**Confidentiality:** All personal identifiers will not be taken hence your responses will be kept confidential. Finally, the data will be analyzed anonymously.

**Participation and withdrawal**: Your participation in this study, which will take you about 15 minutes is fully voluntary. You will be free to withdraw from the study at any time or not to answer questions if you do not want to answer.

**Coordinating organization:** Addis Ababa University, College of Health Sciences, Department of Medical Laboratory Sciences

**Persons to contact:** If you have questions/ concerns about this study you can contact;

Mr**.** Teshiwal Deress (Mob: +251921638642, Email: [teshiwalderes@gmail.com](mailto:teshiwalderes@gmail.com))

Dr. Aster Tsegaye (Mob: +251911696085, Email: [tsegayeaster@yahoo.com](mailto:tsegayeaster@yahoo.com))

Mrs. Fatuma Hassen (Mob: +251911418062, Email: [fatumahassen2000@yahoo.com](mailto:fatumahassen2000@yahoo.com))

Kasaw Adane (Mob: +251919171986, Email: [kswadane@gmail.com](mailto:kswadane@gmail.com))

**Study participant consent form**

**Dear participant!** You are selected from the healthcare professionals in the facility. It is your full right to participate in this study; however, your honest answers to these questions will help us to get important data on the status of knowledge, attitude, and practice of healthcare professionals about biomedical waste management and associated factors, so you are kindly requested to give your honest responses and keep participation. Would you willing to participate, please? If your answer is yes encircle 1 and go to the next part.

1. Yes
2. No

**Dear participant!** For the sake of confidentiality, please do not write your name or other personal identifiers on the questionnaire!

**Questionnaire**

**Instruction**

This questionnaire has four sections. Please read each sections’ instruction and questions properly before you answer the question. If you have unclear instruction or question you can ask data collectors or investigators.

**Section 1: Socio-demographic and healthcare facility related questions**

Please encircle your choice code among the given alternatives below.

| **No.** | **Socio-Demographic Variables** | **Answer** |
| --- | --- | --- |
|  | Sex? | 1. Male 2. Female |
|  | Age in full years? | __________years |
|  | What is your level of education? | 1. MSc or Medical specialist 2. BSc degree 3. Diploma |
|  | In which healthcare facility are you working now? | 1. Hospital 2. Health center 3. Clinic |
|  | What is your job category? | 1. Medical doctor 2. Nurse 3. Midwifery 4. Medical laboratory 5. Health officer |
|  | In which department/ section are you working now? **(More than one answers are possible)** | 1. Outpatient department 2. Ward 3. Laboratory room 4. Emergency room 5. Others (specify) _____ |
|  | How much is your work experience as a healthcare professional? | __________Years |
|  | How much is your working hours per day on your profession? | __________Hours/day |
|  | From where do you get information about biomedical waste management? **(More than one answers are possible)** | From guideline  From training  From friends  Others (specify)_____ |
|  | Have you ever taken training in biomedical waste management or related issues? | 1. Yes 2. No |
|  | Have you taken hepatitis B virus vaccine? | 1. Yes 2. No |
|  | Is there biomedical waste management guideline in your department/ working section? | 1. Yes 2. No 3. Not sure |
|  | Is there biomedical waste management committee at the facility? | - - - 1. Yes       2. No       3. Not sure |
|  | Have you ever encountered any sharp /needlestick injury in the last 12 months? | 1. Yes 2. No |
|  | Are there sufficient quantity gloves in your facility? | 1. Yes 2. No |
|  | Are all 3 types of bins (black bin, yellow bin and safety box) available in your department/ section? | 1. Yes 2. No |

**Section 2: Questions to assess knowledge of healthcare professionals about biomedical waste management and associated factors.**

Please encircle your choice among the possible alternatives for the questions given below in the table.

| No. | Questions to assess healthcare professionals’ knowledge | Answer | | |
| --- | --- | --- | --- | --- |
|  |  | **Yes** | **No** | **Not**  **Sure** |
|  | Does your facility generate biomedical wastes? | 1 | 2 | 3 |
|  | Do you know about biomedical waste management? | 1 | 2 | 3 |
|  | Is there any health hazard associated with biomedical wastes? | 1 | 2 | 3 |
|  | Is needle-stick or sharp injury a concern? | 1 | 2 | 3 |
|  | Does wearing personal protective equipment reduce the risk of infection? | 1 | 2 | 3 |
|  | Are all biomedical wastes biologically hazardous (infectious)? | 1 | 2 | 3 |
|  | Are items contaminated with body fluids considered as biomedical wastes? | 1 | 2 | 3 |
|  | Do you know about color coding segregation of biomedical wastes? | 1 | 2 | 3 |
|  | Should infectious biomedical waste containers be labeled with a biohazard symbol? | 1 | 2 | 3 |
|  | Should biomedical wastes be segregated into different categories at the point of generation? | 1 | 2 | 3 |
|  | Does disinfection of infectious biomedical wastes decrease infection transmission? | 1 | 2 | 3 |
|  | Do we need to close biomedical waste containers while transport? | 1 | 2 | 3 |
|  | Do we need to secure stored biomedical wastes awaiting treatment and disposal? | 1 | 2 | 3 |
|  | Do you know about biomedical waste disposal methods? | 1 | 2 | 3 |

**2.1 Please select and encircle your choice among the given alternatives below**

1. What is the maximum time of infectious biomedical wastes can be stored before treatment or disposal?
   - - 1. 24 hours
       2. 48 hours
       3. 72 hours
       4. I don’t know
2. Which one of the following is an internationally accepted symbol for biohazards?
   - - 1.
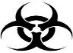

       2.
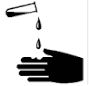

       3.
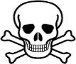

       4.
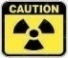

3. What type of biomedical waste should be disposed of in a yellow bin?
   - - 1. General waste
       2. Infectious waste
       3. Another type of waste
       4. I don’t know
4. What type of biomedical waste should be disposed of in a black bin?
   - - 1. General waste
       2. Infectious waste
       3. Another type of waste
       4. I don’t know
5. Where should medical supplies capable of causing puncture/cut to be disposed of?
   - - 1. Black bin
       2. Yellow bin
       3. Safety box
       4. I don’t know
6. How should maximum full be the safety box containing sharp medical supplies?
   - - 1. ½ full
       2. 3/4 full
       3. Full
       4. I don’t know
7. According to World Health Organization guideline, what is the maximum delay to start HIV post-exposure prophylaxis?
   - - 1. 24 hours
       2. 48 hours
       3. 72 hours
       4. I do not know

**Section 3: Questions to assess attitudes of healthcare professionals’ attitude about biomedical waste management and associated factors.**

Based on the following scale of measurement 1-5 ((1=Strongly Disagree (SD); 2=Disagree (D); 3= Neutral (N); 4= Agree (A) and 5=Strongly Agree (SA)); please read each statement and select your answer from the given alternatives below.

| No. | Attitude questions on biomedical waste management | SD | D | N | A | SA |
| --- | --- | --- | --- | --- | --- | --- |
|  | Improperly managed biomedical wastes may cause infection | 1 | 2 | 3 | 4 | 5 |
|  | Proper biomedical waste handling is an issue and a matter of concern | 1 | 2 | 3 | 4 | 5 |
|  | Safe bio-medical waste management is an issue involving a teamwork | 1 | 2 | 3 | 4 | 5 |
|  | HIV may be transmitted through bio-medical wastes | 1 | 2 | 3 | 4 | 5 |
|  | HIV post-exposure prophylaxis can help to prevent the development of HIV infection | 1 | 2 | 3 | 4 | 5 |
|  | Hepatitis B virus can be transmitted through bio-medical wastes | 1 | 2 | 3 | 4 | 5 |
|  | Hepatitis C virus can be transmitted through biomedical wastes | 1 | 2 | 3 | 4 | 5 |
|  | Biomedical wastes do not transmit any infectious diseases | 1 | 2 | 3 | 4 | 5 |
|  | Biomedical wastes should be segregated at the point of generation | 1 | 2 | 3 | 4 | 5 |
|  | Biomedical waste segregation facilitates safe handling of wastes | 1 | 2 | 3 | 4 | 5 |
|  | Labelling biomedical waste containers do not add value to waste management | 1 | 2 | 3 | 4 | 5 |
|  | Proper biomedical waste disposal is important to prevent infection transmission | 1 | 2 | 3 | 4 | 5 |
|  | Biomedical waste disinfection can reduce the chance of contracting the infection | 1 | 2 | 3 | 4 | 5 |
|  | Wearing personal protective equipment helps to reduce the risk of infection | 1 | 2 | 3 | 4 | 5 |
|  | Biomedical waste management adds extra burden of work | 1 | 2 | 3 | 4 | 5 |
|  | Biohazardous wastes should be disinfected before disposal | 1 | 2 | 3 | 4 | 5 |

**Section 4: Questions to assess the practices of healthcare professionals about biomedical waste management and associated factors.**

For the following questions, please encircle your choice among the given alternatives.

| No. | Practice related variables | Response | | Remark |
| --- | --- | --- | --- | --- |
|  | Do you use visual aid/ instructions for biomedical waste segregation in your department/ section? | Yes | No |  |
|  | How often do you use gloves while you are working with/handling of biomedical wastes? | Always  Sometimes  Never | |  |
|  | How often do you wear a gown while you are working with/handling of biomedical wastes? | Always  Sometimes  Never | |  |
|  | Do you label biomedical waste containers? | Yes | No |  |
|  | Do you segregate biomedical wastes according to their type at the point of generation? | Yes | No |  |
|  | If yes on question 408, do you follow color coding segregation? | Yes | No | If No, stop |

1. Where do you put non-infectious wastes like paper, plastic and other supplies which are not contaminated with body fluids?
2. Black waste bin
3. Yellow waste bin
4. Other (specify)______
5. Where do you put infectious wastes like cotton, gauze, and other items contaminated with blood or other body fluids?
6. Black waste container/ bin
7. Yellow waste container/ bin
8. Other (specify)______
9. Where do you put body fluid contaminated sharp medical supplies?
10. Safety box
11. Black plastic bin
12. Yellow plastic bin
13. Other (specify)__

**Dear Participant Thank You for Your Participation!**

**Healthcare Facility Identification Code No.______**

**Study Participant Identification Code No.____**

**Study participant observational checklist**

Observation checklist to assess healthcare professionals’ practice while they were providing healthcare delivery services at Debre Markos town healthcare facilities.

The data collector should observe healthcare professionals on the use of personal protective devices, waste segregation bins, waste segregation bin labelling and status bins whether they are overfilled during the time of observation and tick the appropriate alternative code in the table given below.

| No. | Activities to be observed | Answer | | Remark |
| --- | --- | --- | --- | --- |
|  |  | **Yes** | **No** |  |
|  | Does he/she use gloves while handling/ working with biomedical wastes? | 1 | 2 |  |
|  | Does he/she wear a gown while handling/ working with biomedical wastes? | 1 | 2 |  |
|  | Is there yellow biohazardous waste disposal bin in the section? | 1 | 2 | If No, skip to Qn No 5 |
|  | If yes, on Qn No 3 does it contain only infectious wastes? | 1 | 2 |  |
|  | Is there black biomedical waste disposal bin in the section? | 1 | 2 | If No, skip to Qn No 7 |
|  | If yes, on Qn No 5 does it contain only non-infectious wastes? | 1 | 2 |  |
|  | Is there a biohazard symbol labeled safety box in the section? | 1 | 2 |  |
|  | Does he/she segregate biomedical wastes according to their category? | 1 | 2 |  |
|  | Are all available waste container bins clearly labeled? | 1 | 2 |  |
|  | Is there more than 3/4 filed infectious waste container in the department/ section? | 1 | 2 |  |

Healthcare Facility Identification Code No. ___

**Healthcare facility observational checklist**

Healthcare facility observation checklist to assess real biomedical waste management practices of the healthcare facilities at Debre Markos town.

The data collector should observe the healthcare facility on waste storage and time of storage, treatment method, protection of treatment/disposal site, and waste disposal mechanisms.

| No | Observation parameters | Answer options | Remark |
| --- | --- | --- | --- |
|  | What biomedical waste storage method the facility used? | 1. On-site storage room |  |
|  |  | 1. Puncture resistant storage containers |  |
|  |  | 1. Other specify |  |
|  | Is there infectious waste stored for more than two days in the facility? | 1. Yes |  |
|  |  | 1. No |  |
|  | Does the facility use on-site biomedical waste treatment methods? | 1. Yes | If No, skip to 6 |
|  |  | 1. No |  |
|  | What biomedical waste treatment method the facility used? (Multiple answers are possible) | 1. Incineration |  |
|  |  | 1. Sterilization |  |
|  |  | 1. Chemical |  |
|  |  | 1. Burning |  |
|  |  | 1. Other (specify)_____ |  |
|  |  | 1. No |  |
|  | Are waste treatment/disposal sites protected from unauthorized access? | 1. Yes |  |
|  |  | 1. No |  |
|  | Does the facility use on-site bio-medical waste disposal methods? | 1. Yes | If no, stop |
|  |  | 1. No |  |
|  | What type of waste disposal method used? | 1. Ash Pit | Multiple answers are possible |
|  |  | 1. Needle pit |  |
|  |  | 1. Pit burial |  |
|  |  | 1. Other (specify)____ |  |
